# Training of medical and paramedical personnel in burn care and prevention

**DOI:** 10.4103/0970-0358.70734

**Published:** 2010-09

**Authors:** Tom Potokar, Shariq Ali, Redouane Bouali, Monica Walusimbi, Shobha Chamania

**Affiliations:** Welsh Centre for Burns & Plastic Surgery and Swansea University School of Medicine, Swansea, Wales, UK; 1 Princess Alexandra Hospital, Essex, UK; and Primary Trauma Care and Burns Centre, Dow University of Health Sciences, Karachi, Pakistan; 2 Division Chair Critical Care, University of Ottawa, Canada; and Ministry of Health and Long Term Care, Ontario, Canada and Canadian Patient Safety Institute; 3 Burns & Plastic Surgery Unit, Mulago National Referral Hospital, Uganda; 4 Choithram Hospital & Research Center and Burn Care Foundation, Indore, Madhya Pradesh, India

**Keywords:** Burn care, training, knowledge, skills, attitudes

## Abstract

This paper discusses the requirements for training in burn care within a resource limited environment, what is currently practiced and goes on to suggest a strategy for effective delivery of education and training.

## INTRODUCTION

**Figure 1:**
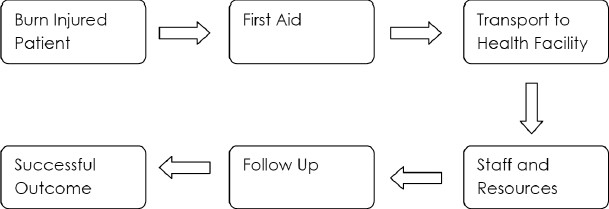
Training of staff in the context of overall burn care

In order to achieve better outcomes in burn injuries, there needs to be a significant change in the whole process of managing burns patients. It is futile to address just one aspect of burn care without regard to other areas; burn care and prevention is above all a team activity and in the same way that no one member of a team should be ignored, no one aspect of burn care should be ignored. Training of staff is critical and is what this article will focus on, but let us firstly put this in the context of overall burn care [Figure 1].

At each stage in the above process, training is important, but needs to be tailored to the specific local conditions and levels of existing knowledge. Prevention is primarily a concept that needs to be understood and acted upon by the general public as is the first-aid treatment. However, in order to do this we must have champions who can motivate and activate people not unlike political agitators, they must be prepared to dynamise the public and get their involvement. They must also have the necessary knowledge in devising prevention campaigns and the means with which to accurately assess their effectiveness. Many patients live in rural areas far from health facilities, particularly health facilities with training in management of burns. Ensuring patients’ arrival in a safe and stable fashion is vital, and management of the early hours of a burn-injured patient must focus on those who are going to be looking after the patient for the initial period.

The majority of burns patients are not treated in specialist centres,[1] and therefore a training campaign should target those with limited knowledge of burn care as the greatest number of patients in total are in district hospitals and rural health facilities. We will concentrate on this training, but without adequate resources, knowledge is a frustration. A huge impact could be made on burn care with relatively simple low cost and easily available interventions. Follow-up is important to convince staff that their efforts produce results and to provide evidence of the health and socio-economic benefits of appropriate care. If the end result is a better outcome for patients, it is not only beneficial on an individual level but also on a community level and the best advocates for improved burn care are those people who have benefitted as a result of it.

### Evidence-based assessment

The first stage in planning an educational program is to undertake an assessment of what currently exists, with particular consideration to who is getting trained, what are they being trained in, how they are being trained, by whom, where and when and why (in other words what are the objectives) [Table 1].

**Table 1:** Typical current training

	*Current situation*	*Ideal situation*
Who	Medical doctors in specialist units	Nurses, general surgeons and general practitioners, healthcare assistants
What	Surgical care	Essential Burn Care
How	‘Apprenticeship’ Visiting specialists	Mixed format with participation and problem-solving approach based on local resources
By whom	Senior surgeons	Local staff with knowledge and commitment to improving burn care (multi-professional)
Where	Big cities	District hospitals and rural areas
When	Ad hoc	Regularly (e.g. twice yearly)
Why	Address needs of major burn- injured patients in well-resourced centres	Majority of patients with mild and moderate burns having little access to specialist centres.

### Needs-based training

Needs-based training implies training the appropriate people to the appropriate level to ensure they have the necessary knowledge, skills and behaviour to optimise their patients’ outcome, provided with adequate resources. This may often necessitate reassessing the role of individuals and requires an open mind on the part of the trainer and the trainee. For example, it is entirely appropriate to teach nursing staff the basics of splinting, physiotherapy and basic rehabilitation as most health facilities will not have the luxury of specialist therapists.

Traditional training programs have tended to focus on didactic material aimed at elevating the level of knowledge in trainees. However, transitory knowledge transfer in itself is not sufficient; it needs to be sustained, which should lead to a change in behaviour and practice with a demonstrated improvement in outcomes. The required change in attitude and behaviour cannot be overemphasised, and for this reason any training curriculum should include activities based on communication skills, motivation, team building and leadership as well as dealing with poorly performing staff. Simulated exercises and rehearsing real-life situations can improve not only retention of knowledge, but also lead to a change in attitudes, following reflection of the experience.[2] Health professionals have a lot to learn from the corporate sector that has studied and developed theories on implementing performance improvement for many years.

If we take a utilitarian approach to burn care, with the aim of doing the most good for the most people, it is evident that spending huge amounts on trying to salvage the few patients with very serious, almost invariably fatal burns, is not the best use of limited resources. Rather we should concentrate at providing the best training (and therefore care) on the bulk of the burden of burn disease that is patients with survivable mild and moderate burns, especially children, who currently have unacceptable outcomes.[3] This then leads us on to the questions of who should be trained, by whom, when, where etc. and we will now look at this in more detail.

#### Who should be trained?

Training in essential burn care should be delivered to those who have access to and see burns patients, who are not going to be referred on to specialist units, either due to lack of transport, lack of finances or for other reasons. Training in advanced burn care should be directed towards those dealing with more serious burn injuries in specialist centres. However, this should not just be for doctors, but critically for nurses and therapists as well.

#### By whom?

To be effective, the quality of training is more important than the qualifications of the trainer. Excellent communication is a priority and whenever possible training should be conducted in the language of the trainees. A listening approach, with the ability to understand the practical day to day problems is required and the trainer should be able to work with participants in any course rather than just preach them. The role of nurses and therapists in training cannot be overemphasised. For advanced burn care, specialists from other countries can add interest and different perspectives so long as they appreciate the constraints of the environment that the trainees are in.

#### What should be taught?

Essential Burn Care is the foundation for all burn care[3] It provides the necessary skills to enable accurate assessment and management of serious burns and the immediate stabilisation and preparation for transfer. However, perhaps more importantly it provides a structured approach to comprehensive basic management of burns including nursing care, positioning, splinting, nutrition, pain management, rehabilitation and emergency surgical procedures such as decompression. Basic Burn Care is a step down from this and covers the rudimentary material required by staff dealing with minor burns on an infrequent basis and the occasional stabilisation of a major burn prior to transfer. Didactic teaching and specific examples are useful in this situation rather than too much background theory.

Advanced Burn Care should be for specialist units and include more emphasis on critical care, operative interventions and post-operative care, development of clinical policies such as antibiotic guidelines and management of the non-survivable burn.

#### How?

Purely didactic training in the form of lectures is not efficient and at best leads to a temporary increase in knowledge. Trainees (medical or paramedical) need to be involved and participating in a learning environment. There is much evidence to suggest that different people learn in different ways; for some visual modalities are best, for others, auditory and others learn best by hands on practice.[4] Using a mixed approach will produce the best results, and specific case-related scenarios that are encountered bring a sense of relevance to the topic.

#### Where?

To reduce costs in terms of travel, accommodation etc., it is best to conduct training in the trainees’ environment whenever possible. This also allows the trainers to appreciate the environment in which burn care has to be delivered by the trainees. Overcrowded wards, with a lack of nurses and limited access to theatres, is part of the reality of burn care and cannot be ignored. Whilst this might be part of the problem, it also has to be looked at as part of the solution. There will be no magic bullet, no sudden influx of resources or funding, and therefore it is a case of training in aspects of burn care that can make a difference at low cost and within existing facilities. This means more emphasis on teamwork and building up a core of committed staff to act as a ‘*micro-burns team*’, who can be motivated to be advocates for their burn patients.

#### When?

‘Tomorrow is too late’ is the motto of interburns (International Network for Training, Education and Research in Burns) and highlights the fact that no time can be wasted. The frequency of training has to take into account the realistic possibilities. Ideally, there should be at least annual training in Essential Burn Care for anyone dealing with burns in a non-specialist environment. The program for those in specialist facilities or who regularly see large numbers of burns is different. In this instance a more formal Advanced Burn Care program should be put in place. For career burns doctors this could be in the format of a postgraduate diploma completed over a number of years. Three month intensive fellowships should be provided in centres of excellence, and particularly important there should be a specific six month specialised training course for registered nurses and /or therapists.

#### Why?

The need for training in burn care is self evident, but what is critical is to appreciate the multidisciplinary care of burns patients and the epidemiology of burn injuries. Children with survivable burns most often end up with the worst scars and contractures and focussing efforts on reducing the burden of disease in this group will have huge personal and societal benefits.

#### Barriers and facilitators

One of the key factors before planning any training program is proactively assessing the various barriers and facilitators to implementation. There are a whole host of factors that can not only contribute to the effectiveness of a training program, but also to its failure.

These factors can be stratified into personal, environmental, financial, cultural, organisational and political. Table 2 suggests some of the potential barriers and facilitators to implementing training in Essential Burn Care to non-specialist staff in rural and district hospitals.

**Table 2:** Barriers and facilitators to burn training

	*Barriers*	*Facilitators*
Personal	Lack of interest	Motivated local ‘champion’
	No perceived benefits from undertaking training	Pre-existing burn team
Environmental	Lack of washing, dressing area, inability to maintain basic hygiene	Ability to modify environment (e.g. separate area for burns patients, dressing room, dedicated theatre time etc.)
Financial	Inability to finance training	Dedicated funds for training
Cultural	Ingrained attitudes that burns are not treatable and inevitably cause scars and contractures	Public awareness and support Staff and patient awareness that appropriate care can improve results
Organisational	Lack of support from hospital management	Supportive, forward thinking hospital management
	No motivation to change existing situation	Commitment to improved services and quality of care
Political	Funding redirected towards expensive equipment	Legislation to ensure certain standards are achieved and adhered to

Understanding these various barriers and facilitators is critical in program planning, as this will allow appropriate program design.[5] It will also be beneficial to discuss these factors during any training program and work together to design solutions to overcoming barriers. For example, if staff feel there is no perceived benefits from training in burn care because ‘all the patients die or get horrible scars anyway’, then this attitude needs to be discussed at the start of any training session. Positive examples can be given of excellent results from similar facilities, which brings not only benefits to patients, but will also raise the status of the staff. What is preferable? A neglected patient staying in hospital for many months, a constant reminder of the inadequacies and failings of medical and nursing care, or a patient discharged with healed wounds, good function and an advocate for the care they received?

### Evaluation

Training must be evaluated, both in the short term and the long term. This is undoubtedly a difficult task especially for long-term evaluation, but is critical to ensuring that the resources spent on training are being used effectively. Immediate post-training assessment will give an indication of temporary knowledge transfer, but the goal should be a sustained knowledge transfer coinciding with behaviour change and improved outcomes. Knowledge attainment is the easiest to assess as this can be done with short questionnaire assessments, direct feedback and by use of examinations. Behaviour change, however, is much more difficult to assess, although there are proxy methods such as looking at outcomes and if they have improved, inferring from this that there has been a change in behaviour.[6] This is certainly an area where more research is needed, particularly in low and middle income countries. Outcomes such as morbidity and mortality require a robust and reliable data collection system and follow-up, which is notoriously difficult in resource-poor environments. Any changes in mortality may also take some time to appear. Again, for morbidity, some proxy methods, while not ideal, can be used. For example, we know that appropriate splinting and positioning limits contracture formation; therefore, a demonstrated increase in the number of patients correctly splinted and positioned is likely to lead to an improvement in outcomes. Ideally, it would be possible to develop comprehensive follow-up data and analysis, but realistically this is extremely difficult to do except perhaps in some of the specialist centres.

Triangulating evaluation by assessing different aspects can help to provide a clearer picture, for example, looking at direct knowledge retention through examination, behaviour change through an audit of antibiotic usage or rates of skin grafting and morbidity through a proxy measure such as length of stay.

### Strategy

The overall strategy then for training in burn care for medical and paramedical staff should be based on a needs-based assessment and tailored to take into account the local barriers and facilitators to implementation of best practice, and reflect the epidemiology of burn injuries. A suggested strategy is suggested below:

Non-specialist doctors, nurses and paramedical staff with limited exposure to burn patients

Annual refresher course in basic burn care.

Learning outcomes

- Ability to safely manage major burns in first few hours and stabilise to transfer to specialist unit
- Ability to provide first aid and teach basic first aid
- Ability to manage minor burns using simple dressings, splinting and positioning

#### Non-specialist doctors, nurses and paramedical staff with regular exposure to burn patients

Annual refresher course in essential burn care.

Learning outcomes

- Understand priorities of burn management (wound care, nutrition, therapy etc)
- Ability to rapidly and accurately assess burn patient needs
- Ability to resuscitate and stabilise patients
- Ability to manage burn wounds and their complications, infection, scars etc
- Perform within a multidisciplinary team
- Skills to undertake emergency surgical procedures such as escharotomy and decompression (medical staff only)
- Skills to perform excision and grafting of small deep burns over critical areas such as joints (medical staff only)
- Ability to undertake very basic reconstruction such as contracture release and skin graft or Z plasty, Y–V plasty. (medical staff only)
- Understand and apply burn prevention strategies
- Understand and apply basic burn rehabilitation

#### Specialist staff in burn facilities

Ongoing advanced burn care

Learning outcomes

- Essential burn care (as above)
- Critical care of major burns
- Ability to manage sepsis within high dependency / critical care unit
- Tangential excision of moderate-sized burns (medical staff only)
- Use of allograft (medical staff only)
- Management of special types of burn for example high-voltage electrical, chemical
- Advanced surgical reconstruction (medical staff only)

At all levels it is imperative to stress the importance of prevention: primary prevention in reducing the number of injuries, secondary in reducing the severity of injury (e.g. through appropriate first aid) and tertiary through reducing the complications of burn injuries.

## SUMMARY

To be effective, training in burn care needs to target all those who are involved in managing burns and to stress the role of the ‘burn team’. The approach must go beyond pure transfer of knowledge and address the skills, attitudes and behaviour of staff and the necessity of implementing best practice within the constraints of available resources.
